# Perceived support and AI literacy: the mediating role of psychological needs satisfaction

**DOI:** 10.3389/fpsyg.2024.1415248

**Published:** 2024-06-14

**Authors:** Yanyan Shen, Wencheng Cui

**Affiliations:** ^1^Faculty of Education, Shaanxi Normal University, Xian, Shanxi, China; ^2^School of Education, Minzu University of China, Beijing, China

**Keywords:** artificial intelligence literacy, psychological needs satisfaction, self-determination theory, teacher support, technical support

## Abstract

Artificial Intelligence (AI) exerts significant influence on both professional and personal spheres, underscoring the necessity for college students to have a fundamental understanding of AI. Guided by self-determination theory (SDT), this study explores the influence of psychological needs satisfaction on AI literacy among university students. A cross-sectional survey involving 445 university students from diverse academic backgrounds was conducted. The survey assessed the mediation effect of students’ psychological need satisfaction between two types of support—technical and teacher—and AI literacy. The results indicate that both support types positively influenced the fulfillment of autonomy and competence needs, which subsequently acted as mediators in enhancing AI literacy. However, the satisfaction of relatedness needs did not mediate the relationship between the types of support and AI literacy. Unexpectedly, no direct association was found between the two forms of support and AI literacy levels among students. The findings suggest that although technical and teacher support contribute to fulfilling specific psychological needs, only autonomy and competence needs are predictive of AI literacy. The lack of direct impact of support on AI literacy underscores the importance of addressing specific psychological needs through educational interventions. It is recommended that educators provide tailored support in AI education (AIEd) and that institutions develop specialized courses to enhance AI literacy.

## 1 Introduction

Our lives and interactions with the external world are being transformed by artificial intelligence (AI) (Kong et al., 2021; Southworth et al., 2023). It is extensively employed in educational settings, especially within colleges and universities (Chu et al., 2022). Previous research on artificial intelligence in education (AIEd) has primarily focused on its utilization in higher education. This encompasses domains such as evaluation, prediction, AI assistants, autonomous tutoring systems, and the management of student learning (Crompton and Burke, 2023). Conversely, empirical research on the AI literacy of university students, particularly those engaged in AI learning at the tertiary educational level, remains scarce (Laupichler et al., 2022). AI literacy entails the ability to effectively recognize, utilize, and assess AI-related products while adhering to ethical standards. It parallels other critical literacies, such as computer literacy and digital literacy (Long and Magerko, 2020; Ng et al., 2021a,b). The scope of AI literacy for non-experts extends beyond computer experts and designers, encompassing students in humanities, social sciences, and other disciplines (Laupichler et al., 2022). Students’ AI literacy significantly varies due to differences in their educational backgrounds or prior experiences (Hornberger et al., 2023). High-quality AI curricula should allow educators to recognize the unique cognitive capabilities of each student while meeting their specific needs (Chiu et al., 2021; Xia et al., 2022). Consequently, it is crucial to integrate the psychological dimension, specifically Self-Determination Theory (SDT), into the analysis of AI literacy.

Based on SDT, when individuals’ basic psychological needs are satisfied, it enhances their intrinsic drive and supports their learning behaviors. The demands that are required include autonomy, competence, and relatedness (Ryan and Deci, 2017). The usage of SDT has been employed in the design of AI courses and to enhance students’ learning competency (Xia et al., 2022, 2023a,b). The proponents of the SDT have also stressed the importance of directing subsequent studies towards the creation of more motivating and effective educational technologies to further improve students’ academic achievements (Ryan and Deci, 2020).

AI literacy for non-experts does not require individuals to be experts in fundamental AI theory or development. Conversely, individuals who adeptly and judiciously use AI products are considered to possess AI literacy (Wang et al., 2023). Consequently, in this study, predictors of AI literacy have been identified as technological conditions and teacher instruction in AIED, rather than more complex mechanisms. Previous research on SDT has concentrated on teacher-student interactions, particularly teacher support (Chiu, 2022, 2023). Recently, numerous studies have begun to explore the role of technological elements in meeting the inherent needs outlined in SDT (Chiu, 2021; Chiu et al., 2022). However, the primary focus of these studies has been on K-12 settings rather than higher education. Additionally, to our knowledge, no studies based on SDT have examined students’ AI literacy. Nevertheless, it is essential for higher education students to enhance their AI literacy, as they will encounter AI applications in both their personal and professional lives (Kong et al., 2022). Collaboration and cooperation with AI are crucial for adapting to the rapidly evolving work environment, especially for individuals who lack innate familiarity with digital technology from a young age (Bennett et al., 2008). Consequently, this study investigates how satisfaction with needs mediates the relationship between two forms of support and AI literacy within the framework of SDT.

## 2 Literature review

### 2.1 Self-determination theory and artificial intelligence literacy

SDT provides a conceptual framework for understanding the development of AI literacy in individuals (Chai et al., 2022; Xia et al., 2022, 2023a,b; Bergdahl et al., 2023). SDT posits that individuals have three psychological needs: autonomy (ownership and control over one’s actions and decisions), competence (successfully completing challenges and tasks), and relatedness (forming close and meaningful relationships with others) (Ryan and Deci, 2020). When students’ three needs are met during their educational journey, they are more likely to actively engage in acquiring AI-related knowledge, exploring AI technologies, and attempting practical applications (Chai et al., 2022; Xia et al., 2023a,b). Positive attitudes toward learning, coupled with corresponding actions, lead to a gradual enhancement in AI literacy, encompassing knowledge mastery, skill development, and ethical concept formation (Chai et al., 2021; Kong et al., 2022).

AI literacy typically entails the ability of individuals to understand, use, monitor, and critically reflect on AI applications, without needing to develop their own AI models (Laupichler et al., 2022). The Technology Acceptance Model (TAM) (Davis, 1989) posits that an individual’s propensity to use a technology is influenced by their perception of its usefulness. This principle extends to AI technologies as well. Individuals’ satisfaction with AI naturally increases when they perceive it as fulfilling their needs and offering practical value (Panagoulias et al., 2024). In the Knowledge-Attitude-Practice (KAP) theoretical model (Kaliyaperumal, 2004), knowledge is defined as the level of understanding concerning a topic or skill. Within the realm of AI, this degree of comprehension is often denoted as AI literacy. Individuals with a greater understanding of AI are better able to identify the potential advantages and benefits of AI technologies and use them effectively to achieve their goals (Serrano, 2022; Xia et al., 2023a). Chai et al. (2022) found that satisfying students’ psychological needs leads to increased pleasure and achievement, which in turn stimulates interest and motivation in learning. This interest and motivation are essential for enhancing AI literacy as they encourage students to actively explore and learn about AI technologies (Zhang et al., 2023). Additionally, Xia et al. (2022) discovered that SDT is pivotal in promoting inclusion and diversity, enhancing learners’ readiness, confidence, and positive attitudes towards AI, reducing anxiety, and boosting intrinsic motivation to learn. Thus, it can be inferred that a positive correlation exists between needs satisfaction and AI literacy.

### 2.2 How teacher and technical support affect needs satisfaction and artificial intelligence literacy

Teachers’ need-supportive actions facilitate the fulfillment of students’ psychological needs (Stroet et al., 2013; Ryan and Deci, 2017). Chiu et al. (2023) employed the concept of needs fulfillment to elucidate the impact of teacher support and student knowledge on the intrinsic motivation to learn, specifically in the context of AI technology. Chen et al. (2021) conducted a study that further validated the existence of beneficial connections between instructional methods and each of the three fundamental psychological needs. The study also highlighted the importance of basic psychological needs as mediators. Although the importance of teacher support is well-documented in SDT research (Vansteenkiste et al., 2020; Chiu, 2021), empirical evidence concerning the impact of instructors on students’ AI literacy is still limited. Casal-Otero et al. (2023) emphasize the critical role of instructors in integrating K-12 AI literacy, based on the limited research available on this topic. To gain a deeper understanding of the forthcoming advancements in AI literacy within the educational domain, it is essential to actively involve more educators (Sperling et al., 2024). AIED is a recently introduced concept in schools (Ng et al., 2021b) that requires active teacher involvement. Chiu et al. (2021) underscore the significance of learner relevance, teacher-student interactions, and adaptability in their AI4future project. These elements are crucial for effective teaching of AI theories and skills. In addition, numerous studies on AI literacy in educational settings focus on developing curricula or courses aimed at enhancing AI literacy (Laupichler et al., 2022). The instructors in these courses offer participants the chance to acquire knowledge about AI (Kong et al., 2021).

In the student learning process, technical support is crucial, especially for online and hybrid courses (Lee et al., 2011; Zheng et al., 2018). Research has shown that technical support can significantly enhance students’ literacy skills. Additionally, it has been found that meeting students’ needs mediates the relationship between technical support and literacy competence (Chiu et al., 2022). Chiu (2022) found that teachers can effectively address students’ psychological needs by integrating technology into their education. In a separate study by Chiu (2022), the focus was on examining how digital support could meet three fundamental psychological needs, thereby increasing student engagement in blended learning. Studies have developed unplugged learning activities to enhance students’ AI literacy independently of computers. These activities include methods such as case studies, role-playing, and storytelling (Julie et al., 2020; Rodríguez-García et al., 2020). However, the use of computing-related learning materials, including technical support, remains crucial for developing AI literacy (Chai et al., 2020; Cavalcanti et al., 2021). It is essential to provide age-appropriate learning materials to students to enhance their understanding of AI concepts and to foster their enthusiasm and interest in learning AI (Ng et al., 2021b). Additionally, Kong et al. (2021) supported learners in problem-solving by developing AI applications during the third stage of their AI literacy program. The findings showed a significant improvement in participants’ self-perceived AI literacy, evidenced by an increase in the mean score from 2.93 before the course to 3.98 after the course.

### 2.3 This study

**Figure 1:**
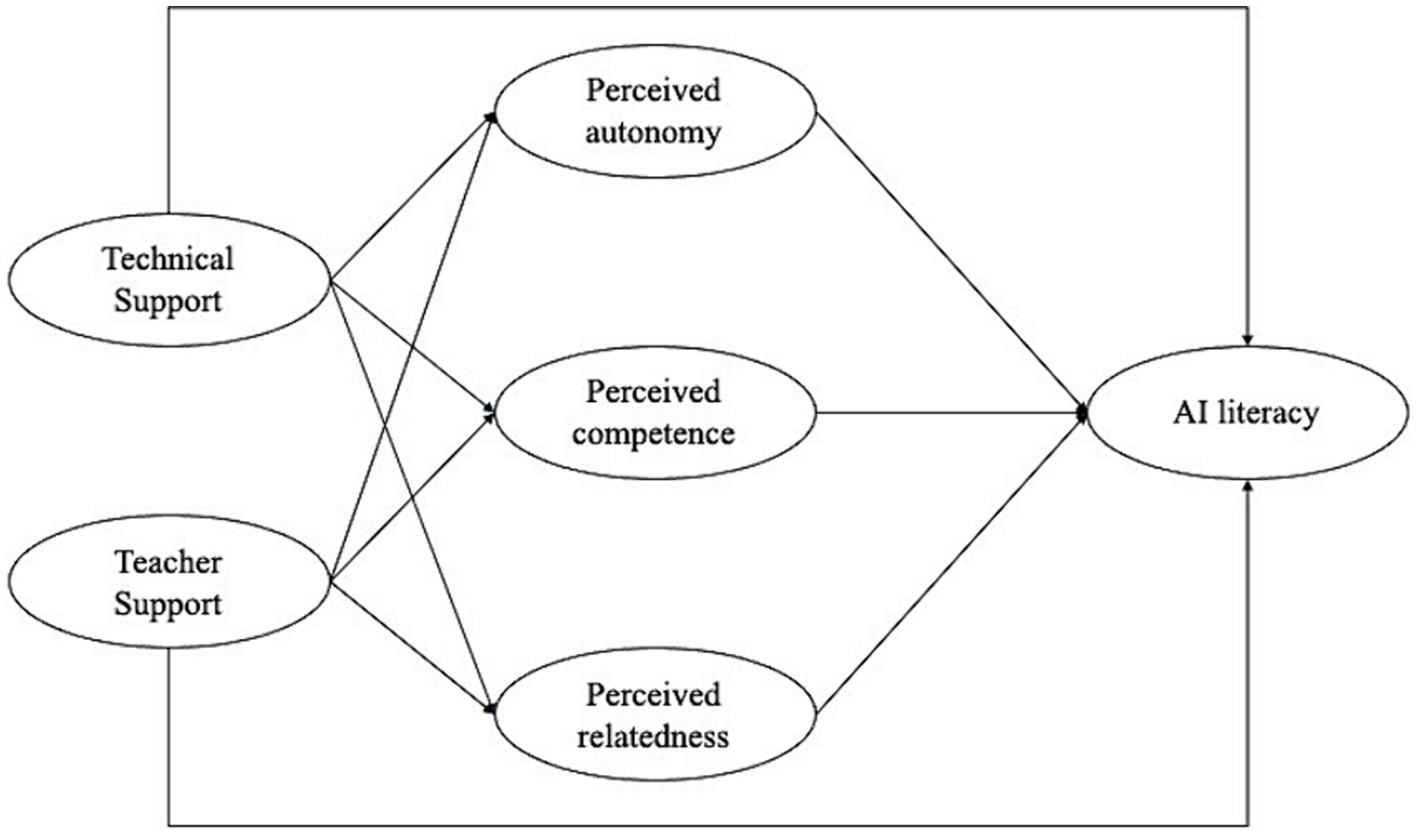
The hypothesized research model.

After reviewing the relevant literature, the current study presents a hypothesized research model with five hypotheses (Figure 1).

*H1*: Technical support will contribute positively to meeting the three needs (autonomy, competence, and relatedness) (H1a, H1b, H1c).

*H2*: Teacher support will make a positive contribution to meeting these three needs (autonomy, competence, and relatedness) (H2a, H2b, H2c).

*H3*: Both types of support (technical support, teacher support) will contribute positively to students' AI literacy (H3a, H3b).

*H4*: The fulfilment of each of the three needs (autonomy, competence, and relatedness) will contribute positively to students' AI literacy (H4a, H4b, H4c).

*H5*: Needs fulfilment (autonomy, competence, and relatedness) will mediate the relationship between both supports and AI literacy (H5a, H5b, H5c).

## 3 Method

### 3.1 Participants and research procedure

Many higher education institutions in China have embarked on “AI + Education” initiatives. Through these efforts, AI provides students with personalized, round-the-clock learning support, intelligent assessments, and feedback. Furthermore, AI aids teachers in managing classrooms, courses, and students, thereby harmonizing the roles of AI technology and educators. This study employs purposive sampling to select participants based on two criteria: (1) Technical Support: We examined whether these institutions have developed comprehensive intelligent teaching platforms that provide students with advanced AI learning tools and resources. (2) Teacher Support: We assessed whether teachers effectively use these platforms in their teaching processes to offer timely guidance and support to students. This includes sharing AI learning resources and guiding students in exploring effective AI learning methods. Through news gathering, website browsing, and online interviews, we selected three universities that met the criteria as candidates for this study. The “AI + Education” practices at these universities do not aim to impart complex AI professional knowledge. Instead, they leverage innovative tools such as intelligent teaching systems, smart classrooms, virtual assistants, and personalized learning platforms to enhance teaching and learning across various disciplines, aligning with the concept of general AI literacy. Additionally, we observed that each institution has distinctive characteristics. For example, institution 1 focuses on training pre-service teachers in using software such as AI PPT and PowToon to create courseware, as well as applications like ChatGPT for intelligent grading. In addition, institution 2 is known for its self-developed intelligent teaching platform, “Xiaoya,” which enables real-time interaction between teachers and students, monitors students’ learning status, and provides timely academic feedback or warnings. Institution 3 emphasizes the integration of professional education and AI technology. For instance, the “AI Sight-Singing and Ear-Training Programmer,” developed by teachers and students, integrates essential teaching resources for conservatory students and allows students to develop learning resources under the guidance of teachers.

From February to March 2024, we conducted an online survey by sharing a QR code for the questionnaire via WeChat and QQ groups. The entire survey process ensured data confidentiality and security. All participants’ personal information and questionnaire data were encrypted and used solely for research purposes. We collected a total of 490 questionnaires, but 45 were excluded due to invalid responses (e.g., linear answers, excessively fast responses, or inaccurate data entry). After meticulous data cleaning, we obtained 445 valid questionnaires, which serve as the foundation for our in-depth analysis and research. Table 1 provides detailed information on the final sample (*N* = 445) for further discussion and study.

**Table 1:** Characteristics of the sample (*N* = 445).

**Profile**	**Category**	**Frequency**	**Percentage (%)**
Gender	Male	92	20.7
	Female	353	79.3
Degree	Junior college student	28	6.3
	Undergraduate	344	77.3
	Master	68	15.3
	PhD	5	1.1
Age	18–22	370	83.1
	23–27	57	12.8
	27–31	10	2.2
	≥ 32	8	1.8
Major	Education	192	43.1
	Literature	128	28.8
	Science	30	6.7
	Management	17	3.8
	Other	78	17.5

### 3.2 Instrument

A four-part questionnaire was used as the primary research instrument for this study. The first section contained demographically relevant questions about age, gender, year of college attendance, and major. The second section assessed students perceived technical support and teacher support in AI education. Part III included questions about AI literacy, including awareness, use, assessment, and ethics. Part IV assessed three perceived needs: perceived autonomy, perceived competence, and perceived relevance. The questionnaire used a 5-point Likert scale, based on the criteria of Brown (2010), ranging from “strongly disagree” to “strongly agree” to measure frequency of use. All the items are attached in the index.

#### 3.2.1 Teacher support

The Teacher Support Scale comprises six items, adapted from the work of Lai (2015). One of the items states that teachers should promote the use of AI apps or products for learning outside the classroom.

#### 3.2.2 Technical support

The Technology Support Scale comprises four items and was adapted from a subscale of the Lee et al. (2011) Student Learning Support Scale. One of the items reads, “I have experienced numerous technical difficulties with AI learning.”

#### 3.2.3 Needs satisfaction

The Need Satisfaction Scale comprises 12 items that encompass three measuring dimensions: perceived autonomy, perceived competence, and perceived relevance. Among these, Hew and Kadir (2016) provided adaptations for the items measuring perceived competence and autonomy. An illustration of a perceived autonomy item is the statement, “I am presented with ample opportunities to exercise my own agency in determining the methods by which I acquire knowledge through my usage of chatbots.” An instance of a perceived competency item is, “Engaging with the chatbot provides me with a feeling of achievement.” Furthermore, Furrer and Skinner (2003) were the source of questions used to measure perceived relatedness. A concrete illustration is, “I experience a sense of bolstering when I use the chatbot for educational purposes.”

#### 3.2.4 Artificial intelligence literacy

The Artificial Intelligence Literacy Scale comprises 12 items that encompass the four measuring aspects of awareness, use, evaluation, and ethical considerations. These dimensions have been developed from Wang et al. (2023). An example of an awareness item is the statement, “I possess the ability to identify the artificial intelligence technologies used in the applications and products that I use.” An illustration of a usage item is, “I am capable of employing an artificial intelligence application or product to enhance my acquisition of knowledge.” An illustration of an evaluation item could be, “I possess the ability to choose the most appropriate AI application or product from a diverse range of options for a specific task.” An instance of an ethical item could be expressed as, “I consistently adhere to ethical principles when applying an AI application or product.”

### 3.3 Data analysis

During the data analysis session, we used two software packages, namely Mplus 8.3 and SPSS 26.0. Initially, we used SPSS 26.0 for descriptive statistics, correlation analyses, and calculating Cronbach’s alpha coefficients. These analyses provide a foundational understanding of the data and ensure the scales’ reliability. Subsequently, we used Mplus 8.3 for confirmatory factor analysis (CFA) and structural equation modeling (SEM). We also employed bootstrapping to test mediation effects. These analyses are crucial for testing the reliability and validity of the measurement models and evaluating the structural relationships between variables.

## 4 Results

### 4.1 Descriptive statistics and reliabilities

The correlation and mean and standard deviation of the measurements are shown in Table 2. The values of kurtosis vary from −0.8 to 0.20, while the values of skewness range from −0.23 to 0.40. Additionally, a *p*-value of 0.05 or lower from the correlation analysis indicated a statistically significant positive relationship between the variables under investigation.

**Table 2:** Descriptive statistics and correlations between the measured variables.

**Variable**	**1**	**2**	**3**	**4**	**5**	**6**	**7**	**8**	**9**	**10**
1. Teacher	1									
2. Tech	0.47**	1								
3. PA	0.40**	0.34**	1							
4. PC	0.50**	0.64**	0.33**	1						
5. PR	0.54**	0.46**	0.43**	0.48**	1					
6. AW	0.46**	0.43**	0.42**	0.46**	0.54**	1				
7. US	0.45**	0.39**	0.44**	0.40**	0.53**	0.68**	1			
8. EV	0.37**	0.37**	0.31**	0.38**	0.41**	0.54**	0.58**	1		
9. ET	0.24**	0.31**	0.24**	0.30**	0.38**	0.35**	0.33**	0.46**	1	
10. AL	0.48**	0.48**	0.45**	0.49**	0.59**	0.82**	0.83**	0.82**	0.69**	1
Mean	3.78	3.82	3.78	3.71	3.74	3.57	3.55	3.69	4.19	3.75
SD	0.51	0.54	0.57	0.58	0.59	0.60	0.62	0.59	0.62	0.48
Skewness	0.34	−0.08	0.03	0.06	0.28	0.15	0.40	0.26	−0.23	0.26
Kurtosis	−0.02	0.20	−0.11	0.03	−0.50	−0.06	−0.09	0.02	−0.80	−0.15

The numbers in diagonal brackets are the coefficients α; *N* = 445.Teacher, teacher support; Tech, technical support; PA, perceived autonomy; PC, perceived competence; PR, perceived relevance; AW, awareness; US, usage; EV, evaluation; ET, ethics; AL, AI literacy.**p < 0.01.

### 4.2 Evaluation of the measurement model

The constructs in the proposed model were subjected to reliability and validity assessments. Table 3 indicates that all CRs exceeded 0.70. To evaluate the internal consistency, we employed Cronbach’s alpha coefficients. The findings demonstrated that all constructs satisfied the requirements established by Fornell and Larcker (1981), as evidenced by Cronbach’s alpha coefficients ranging from 0.71 to 0.87.

**Table 3:** Results for the measurement model.

**Construct**	**Item**	**Factor loading**	**Cronbach’s alpha**	**CR**	**AVE**
Teacher support			0.80	0.81	0.43
	TS1	0.58			
	TS2	0.53			
	TS3	0.74			
	TS4	0.69			
	TS5	0.66			
	TS6	0.68			
Technical support			0.75	0.76	0.46
	TECH1	0.75			
	TECH2	0.73			
	TECH3	0.74			
	TECH4	0.44			
PA			0.76	0.76	0.45
	PA1	0.53			
	PA2	0.69			
	PA3	0.80			
	PA4	0.64			
PC			0.78	0.78	0.47
	PC1	0.70			
	PC2	0.71			
	PC3	0.71			
	PC4	0.62			
PR			0.76	0.76	0.45
	PR1	0.67			
	PR2	0.62			
	PR3	0.74			
	PR4	0.64			
AI literacy			0.87	0.89	0.69
AW		0.96	0.71	0.71	0.46
	AW1	0.64			
	AW2	0.79			
	AW3	0.58			
US		0.95	0.74	0.75	0.51
	US1	0.84			
	US2	0.71			
	US3	0.57			
EV		0.82	0.74	0.74	0.49
	EV1	0.70			
	EV2	0.75			
	EV3	0.64			
ET		0.50	0.82	0.82	0.60
	ET1	0.74			
	ET2	0.82			
	ET3	0.77			

PA, perceived autonomy; PC, perceived competence; PR, perceived relevance; AW, awareness; US, usage; EV, evaluation; ET, ethics.

To assess the convergent validity of the dimensions in the measuring model, we performed a second-order confirmatory factor analysis (CFA) of AI literacy. The results demonstrated a strong fit of the model, as evidenced by the following statistics: *χ*^2^ = 130.20, *χ*^2^/*df* = 2.60, CFI = 0.954, TLI = 0.939, and SRMR = 0.041. Table 3 presents the standardized estimates of factor loading values for all constructs, which varied between 0.44 and 0.86. Despite instances of low average variance extracted (AVE), Fornell and Larcker (1981) proposed that AVE may be a more cautious metric, and that the convergence of construct validity can still be confirmed only based on composite reliability (CR). Since both CRs above 0.70, the convergent validity of the constructs was deemed satisfactory. Overall, the items of the proposed measuring model demonstrated satisfactory levels of reliability and validity.

The results of the discriminant validity evaluation are shown in Table 4. Strong discriminant validity is suggested by the square root of the AVE, which is greater than the correlation between each concept and all other components.

**Table 4:** Discriminant validity.

	**1**	**2**	**3**	**4**	**5**	**6**
1.Teacher support	**0.66**					
2.Technical support	0.50**	**0.68**				
3.Perceived autonomy	0.32**	0.38**	**0.72**			
4.Perceived competence	0.32**	0.38**	0.22**	**0.78**		
5.Perceived relevance	0.43**	0.44**	0.27**	0.24**	**0.71**	
6.AI literacy	0.58**	0.63**	0.48**	0.44**	0.50**	**0.85**

**p < 0.01. The numbers in parentheses on the diagonal are the square root of AVE.

### 4.3 Testing the structural model and hypotheses

With *χ*^2^ = 993.429, *χ*^2^ /*df* = 1.94, CFI = 0.916, TLI = 0.907, and SRMR = 0.053, the structural model demonstrated a satisfactory model fit.

**Figure 2:**
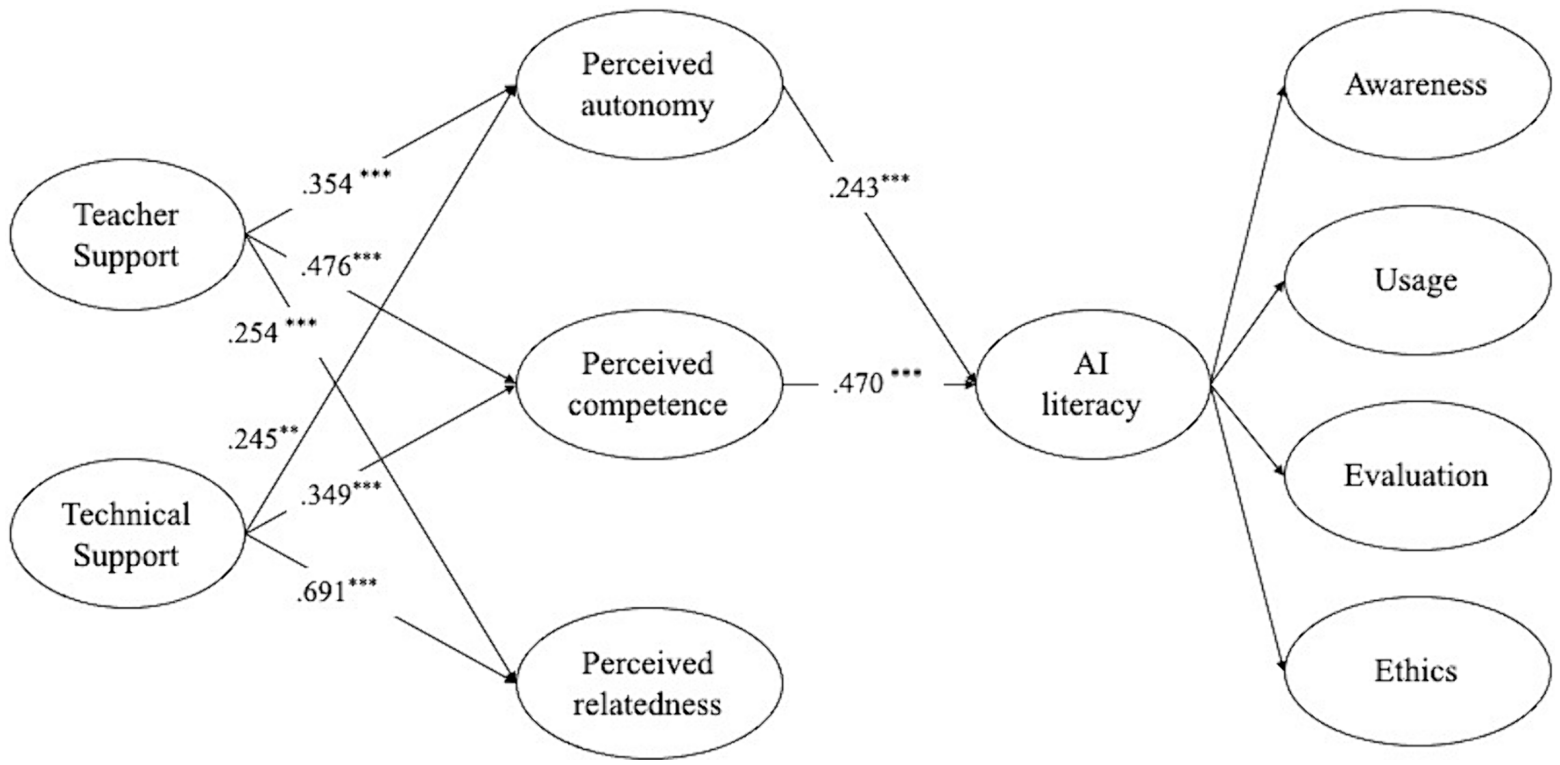
Results for the Structural model.

Figure 2 displays the structural coefficients of the model. The impact of technical support on autonomy (*β* = 0.245, *p* < 0.01), competence (*β* = 0.349, *p* < 0.001), and relatedness (*β* = 0.691, *p* < 0.001) was shown to be significant. Thus, the initial research hypothesis (H1) was confirmed. Teacher support has a substantial and direct impact on autonomy (*β* = 0.354, *p* < 0.001), competence (*β* = 0.476, *p* < 0.001), and relatedness (*β* = 0.254, *p* < 0.001). Thus, the second research hypothesis (H2) was confirmed. There was no significant link between teacher support (*β* = 0.44, *p* = 0.532) or technical support (*β* = 0.75, *p* = 0.593) and students’ AI literacy. Consequently, the third research hypothesis was ignored. Aside from relatedness (*β* = 0.141, *p* = 0.260), both autonomy (*β* = 0.243, *p* < 0.001) and competence (*β* = 0.470, *p* < 0.001) had a notable and direct impact on students’ AI literacy. Thus, H4a and H4b were approved whereas H4c was denied.

To look at the moderating effects of needs satisfaction, we used bootstrapping. To determine the 95% confidence intervals (CIs) for the effects of technical support and teacher support on AI literacy, we used bootstrapping on a random sample of 1,000. This was done to look at how autonomy, competence, and relevance function as mediators.

The indirect impacts of technical support and teacher support on AI literacy were found to have coefficients of 0.32 and 0.35, respectively. These coefficients were accompanied by 95% confidence intervals of [0.133, 0.553] and [0.220, 0.497] for technical support and teacher support, respectively. It is worth noting that the direct effect was not statistically significant. The 95% confidence intervals did not include the value of zero. Both technical support and teacher support were important factors in predicting AI literacy via fulfilling psychological needs. Indirect indicator of proficiency in artificial intelligence. The combined impact of technical support and teacher support on AI literacy was 0.40 and 0.39, respectively. The correlation analysis indicates that the mediation of demands in the distinctive design is mostly driven by autonomy and competence, rather than other less important indirect effects. Hence, our results suggest that the three categories of needs satisfaction had a partial impact on the relationship between technical support and AI literacy, as well as between instructor support and AI literacy (Table 5).

**Table 5:** Standardized total, total, indirect, and direct effects among variables.

**Predictor**	**Mediating/criterion variable**	**Direct effect**	**Indirect effect**	**Total effect**
Technical support	Perceived autonomy	0.25 (*p* < 0.01)	_	0.25
	Perceived competence	0.35 (*p* < 0.001)	_	0.35
	Perceived relatedness	0.69 (*p* < 0.001)	_	0.69
	AI literacy	0.08 (*p* = 0.532)	0.32	0.40
Teacher support	Perceived autonomy	0.35 (*p* < 0.001)	_	0.35
	Perceived competence	0.48 (*p* < 0.001)	_	0.48
	Perceived relatedness	0.25 (*p* < 0.001)	_	0.25
	AI literacy	0.04 (*p* = 0.593)	0.35	0.39
Perceived autonomy	AI literacy	0.24 (*p* < 0.001)	_	0.24
Perceived competence	AI literacy	0.47 (*p* < 0.001)	_	0.47
Perceived relatedness	AI literacy	0.14 (*p* = 0.260)	_	0.14

## 5 Discussion and conclusion

To evaluate students’ AI literacy in ‘AI + Education’ practice, we examined how the fulfilling of psychological demands influences the connection between two forms of support and AI literacy. Our analysis revealed support for six hypotheses (H1, H2, H4a, H4b, H5a, H5b) and rejection of three hypotheses (H3, H4c, H5c), indicating overall support for the theoretical model. Further discussion will be provided below regarding the findings.

Our findings indicate that technical support would have a good impact on fulfilling the three needs (H1). The findings from the survey primarily reflect the students’ subjective opinions of AI educational technology, rather than providing an accurate assessment of the real technological capabilities of AIED. Hence, this study’s examination of perceived technology support is intricately linked to the satisfaction of psychological needs in AIED. The findings of this study agree with prior research that suggests technical support plays a crucial role as a learning resource in meeting students’ needs (Chiu, 2022; Chiu et al., 2022). According to Chiu et al. (2022), when technology-rich environments effectively meet these three requirements, students gain increased autonomy in selecting technology for working with digital resources, an improved sense of competence in using technology ethically for creation and sharing, and a heightened sense of connection to collaboration and communication. Teacher support, which is also a significant indicator of AI literacy, has a positive effect on fulfilling the three demands (H2). Empty text. ‘AI + Education’ practice, as a recent endeavor (Ng et al., 2021b), still requires teachers’ comprehension of students’ personalities and cognitive capacities, as well as their provision of support tailored to their requirements (Chiu et al., 2021; Xia et al., 2022). The conclusions have been corroborated by an extensive Research Topic of prior investigations (Vansteenkiste et al., 2020; Chiu, 2023; Chiu et al., 2023).

Our study discovered that while both supports had a favorable impact on fulfilling the three requirements (H1, H2), only the fulfilment of the autonomy need, and the competence need were shown to be predictors (H4) and mediators (H5) in the relationship between the two supports and AI literacy. However, the satisfaction of relevant demands did not serve as a predictor or mediator in the link between the two. The results match with the research conducted by Chiu et al. (2022) and Xia et al. (2023b), which proposed that the satisfaction of autonomy and competence needs plays a crucial role in learning capacity and cognitive processes. However, the impact of relevance was found to be insignificant. Hence, the presence of technical settings and teacher training that foster autonomy and competence needs can have a favorable impact on students’ AI literacy. The results corroborate the implications of the theory of mind (Langley et al., 2022) in the field of AI, indicating that the relationship between students and AI is predominantly cognitive in nature, rather than emotional.

In contrast to predictions, there was no direct correlation between students’ AI literacy and the two forms of support (teacher support and technical support) (H3). This could be attributed to the limited ability of chatbots in AIED to comprehend intricate expressions (Jeon, 2024). Additionally, individuals without a background in computer science, commonly referred to as “non-experts,” priorities the effectiveness of their interaction with the technology rather than the underneath AI model (Laupichler et al., 2022). Moreover, the field of ‘AI + Education’ involves the integration of AI technology into various academic disciplines. Instructors who are not AI experts may not directly teach AI-specific knowledge to their students, but they can still have an indirect impact on their students’ learning by AI-assisted teaching systems (Ahmad et al., 2022).

In summary, our work has discovered a previously unknown empirical correlation between two forms of support and AI literacy, using the SDT theory. The implications of our findings for ‘AI + Education’ practice is threefold. Current AI-assisted teaching systems may lack maturity, hindering efficient interaction between non-expert learners and the technology (Xia et al., 2023b). Consequently, the role of teachers in AIED remains significant (Chiu et al., 2021). We recommend that educators use AI technology to enhance teaching and learning, while also being mindful of the unique characteristics and cognitive capacities of each student, and providing the necessary support accordingly (Chiu et al., 2021; Xia et al., 2022). By addressing students’ psychological needs, it is possible to foster the development of their AI literacy. However, schools can offer students targeted AI literacy courses due to the interdisciplinary nature of AIEd (Crompton and Burke, 2023). By enhancing their AI literacy, individuals can thrive in their professional attempts and play a significant role in their everyday lives (Kong et al., 2022). Currently, Chinese higher education institutions are not giving sufficient attention to AI literacy instruction, as far as we know. Ultimately, we offer valuable perspectives for educational administrators and artificial intelligence programmers. Our research indicates that the current state of AI technologies mostly focuses on tasks such as classroom management, course administration, and student management. However, these technologies are not yet well linked with instructional content and offer limited learning support for students. Many students lack clarity on the specific knowledge they should acquire through AI systems and how to properly use AI technology to enhance their mastery of knowledge. Consequently, it is essential for higher education institutions to enhance their partnership with technology companies to create more suitable AI products (Benedito Saura, 2018).

## 6 Limitation and future research direction

Based on our survey of three colleges that have implemented ‘AI + Education’ practices, several conclusions have been drawn. It is crucial to acknowledge that variations exist in the implementation of ‘AI + Education’ practices across educational institutions. From utilizing AI tools such as AI PPT and PowToon for pre-service teacher training to developing intelligent teaching platforms like Xiaoya, and integrating professional education with AI technologies, the differences in focus among these institutions underscore the complexity and multifaceted nature of ‘AI + Education’ initiatives. This diversity, although a crucial aspect of our findings, complicates the derivation of generalizable conclusions, as different approaches may yield varying levels of effectiveness and student acceptance. Future research should aim to conduct quasi-experimental studies that control for or isolate the effects of various ‘AI + Education’ implementation methods. Such studies would facilitate a deeper understanding of the specific impact of each method and provide more precise insights into its effectiveness.

Additionally, this study utilized a cross-sectional research design, which somewhat limited our ability to infer causal relationships. Since data Research Topic occurred at a single point in time, we could not identify the dynamic interactions among perceived support, AI literacy, and psychological needs satisfaction, nor their processes of change. This limitation may have compromised the explanatory power of our findings. To address the limitations inherent in cross-sectional studies, future research should consider adopting a longitudinal research design. By collecting data at multiple points in time, researchers can observe trends in the relationships among variables and thereby infer causality more accurately.

Furthermore, this study focused on the basic psychological needs in SDT and explored how these needs mediate the relationship between perceived support and AI literacy. Although this offers a valuable perspective for understanding the psychological processes involved, the study did not consider other potential influences, such as individuals’ prior skill levels, allocation of educational resources, curricular differences, faculty strengths, and learning climates. These factors might similarly influence AI literacy. Future research should expand the current theoretical framework to include additional variables that could affect AI literacy. Subsequent studies could employ a mixed-methods research design that integrates quantitative and qualitative data to more comprehensively understand how these factors interact.

Finally, the gender composition of the sample was predominantly female. This gender imbalance may have influenced the study’s results. We recommend that future studies enhance our findings by including a more balanced gender representation, thereby increasing the generalizability of the results. Future research should also further investigate the impact of gender on the effectiveness and acceptance of ‘AI + Education’ practices. Such investigations could determine whether the observed effects are consistent across genders or if significant differences need to be addressed. We will therefore include detailed gender analyses in future AI literacy studies to more fully explore this aspect.

## Data availability statement

The raw data supporting the conclusions of this article will be made available by the authors, without undue reservation.

## Ethics statement

The studies involving humans were approved by Minzu University of China Shaanxi Normal University Central China Normal University. The studies were conducted in accordance with the local legislation and institutional requirements. The participants provided their written informed consent to participate in this study.

## Author contributions

YS: Conceptualization, Data curation, Formal analysis, Funding acquisition, Project administration, Resources, Validation, Visualization, Writing – original draft, Writing – review & editing. WC: Data curation, Formal analysis, Investigation, Methodology, Software, Writing – original draft.
