# Supplementary material for: Perceived support and AI literacy: the mediating role of psychological needs satisfaction
Source: Front Psychol. 2024 Jun 14;15:1415248. doi: 10.3389/fpsyg.2024.1415248 (PMC11212795; doi:10.3389/fpsyg.2024.1415248)
Supplement: Supplementary file 1 [file Table_1.DOCX]

Supplementary Material

# APPENDIX

| **Items** | **Statements** |
| --- | --- |
| Teacher support (Lai, 2015) | |
| TS1 | My teacher encourages us to use AI for learning outside the classroom. |
| TS2 | My teacher discusses with us how AI could enhance learning. |
| TS3 | My teacher shares with us useful AI resources/sites/tools for learning outside the classroom. |
| TS4 | My teacher shares tips/strategies on how to use AI for learning. |
| TS5 | My teacher often uses AI in their classes. |
| TS6 | My teacher engages us with learning activities that involve the use of AI. |
| Technical support (Lee et al., 2011) | |
| TECH1 | Seeking assistance with AI technology was challenging for me. |
| TECH2 | I knew where to seek help for any AI technology issues. |
| TECH3 | AI technology support addressed my issues promptly. |
| TECH4 | I was confident in accessing AI technology support as needed. |
| Needs satisfaction (Furrer and Skinner, 2003; Hew and Kadir, 2016) | |
| Perceived autonomy (Hew and Kadir, 2016) | |
| PA1 | I feel like I can make a lot of input in deciding how I use the AI applications or products in learning. |
| PA2 | I feel a sense of freedom when using the AI applications or products. |
| PA3 | I have many opportunities with the AI applications or products to decide for myself how to learn. |
| PA4 | I have a say regarding what input I want to learn with AI applications or products. |
| Perceived competence (Hew and Kadir, 2016) | |
| PC1 | I think I am pretty good at learning with the AI applications or products. |
| PC2 | I have been able to learn interesting new knowledge with the AI applications or products. |
| PC3 | I feel a sense of accomplishment from learning with the AI applications or products. |
| PC4 | When I am using AI applications or products, I often do not feel very capable R |
| Perceived relatedness (Furrer and Skinner, 2003) | |
| PR1 | When I learn with the AI applications or products, I feel supported. |
| PR2 | When I learn with the AI applications or products, I feel comfortable. |
| PR3 | When I learn with the AI applications or products, I feel important. |
| PR4 | When I learn with the AI applications or products, I feel valued. |
| AI literacy (Wang et al., 2023) | |
| Awareness | |
| AW1 | I can distinguish between smart devices and non-smart devices. |
| AW2 | I know how AI technology can help me. |
| AW3 | I can identify the AI technology employed in the applications and products I use. |
| Usage | |
| US1 | I can skillfully use AI applications or products to help me with my daily work. |
| US2 | It is usually easy for me to learn to use a new AI application or product. |
| US3 | I can use AI applications or products to improve my work efficiency. |
| Evaluation | |
| EV1 | I can evaluate the capabilities and limitations of an AI application or product after using it for a while. |
| EV2 | I can choose a proper solution from various solutions provided by a smart agent. |
| EV3 | I can choose the most appropriate AI application or product from a variety for a particular task. |
| Ethics | |
| ET1 | I always comply with ethical principles when using AI applications or products. |
| ET2 | I am alert to privacy and information security issues when using AI applications or products. |
| ET3 | I am always alert to the abuse of AI technology. |

PA perceived autonomy, PC perceived competence, PR perceived relevance, AW awareness, US usage, EV evaluation, ET ethics

Furrer, C., and Skinner, E. (2003). Sense of relatedness as a factor in children's academic engagement and performance. *Journal of educational psychology* 95(1)**,** 148. doi: <https://doi.org/10.1177/0272431693013001002>.

Hew, T.-S., and Kadir, S.L.S.A. (2016). Understanding cloud-based VLE from the SDT and CET perspectives: Development and validation of a measurement instrument. *Computers & Education* 101**,** 132-149. doi: <https://doi.org/10.1016/j.compedu.2016.06.004>.

Lai, C. (2015). Modeling teachers' influence on learners' self-directed use of technology for language learning outside the classroom. *Computers & Education* 82**,** 74-83. doi: <https://doi.org/10.1016/j.compedu.2014.11.005>.

Lee, S.J., Srinivasan, S., Trail, T., Lewis, D., and Lopez, S. (2011). Examining the relationship among student perception of support, course satisfaction, and learning outcomes in online learning. *The internet and higher education* 14(3)**,** 158-163. doi: <https://doi.org/10.1016/j.iheduc.2011.04.001>.

Wang, B., Rau, P.-L.P., and Yuan, T. (2023). Measuring user competence in using artificial intelligence: validity and reliability of artificial intelligence literacy scale. *Behaviour & information technology* 42(9)**,** 1324-1337.
